# Extranodal Rosai-Dorfman disease of the kidney

**DOI:** 10.4103/0256-4947.51823

**Published:** 2009

**Authors:** Alireza Abdollahi, Farid A. Ardalan, Mohsen Ayati

**Affiliations:** a From the Department of Pathology, Tehran University of Medical Science, Tehran, Iran; b From the Department of Urology, Tehran University of Medical Science, Tehran, Iran

Lampert and Lennert in 1961 were the first to describe what is now known as Rosai-Dorfman disease (RDD). Subsequently in 1969, Rosai and Dorfman described 4 cases of a disease they called sinus histocytosis with massive lymphadenopathy (better known as RDD).^1^ In 1972 they analyzed 30 additional cases, establishing RDD^2^ as a clinicopathologic entity. RDD generally manifests in children or young adults with massive cervical lymphadenopathy, fever, leukocytosis, an increased erythrocyte sedimentation rate and hypergammaglobulinemia. Other lymphatic groups such as mediastinal, axillary and inguinal lymph nodes can also be affected.^3^ In about 25% to 40% of cases, extranodal sites are also affected. Extranodal RDD lesions may occur with or without lymphadenopathy and may be solitary or multiple.^4^ The concomitant involvement of one or more sites in the same individual is observed in up to 44.7% of cases. Extranodal involvement is often responsible for the most important clinical manifestations of the disease.^5^ Despite its alarming appearance, RDD is considered a benign condition in the majority of cases because of its self-limited course.

We report a rare case in which the patient presented with initial involvement of the left kidney in the absence of nodal involvement.

## CASE

**Figure 1:**
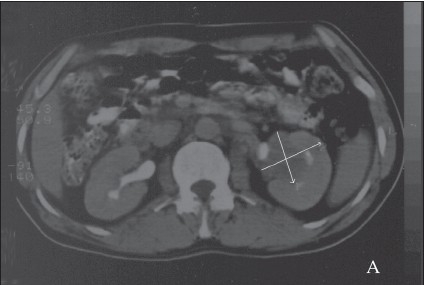
A CT-scan shows a homogenous low-density area involving the left renal sinus that shows distortion and displacement of the pelvicalyceal system without hydronephrosis (arrows demonstrates).

**Figure 2:**
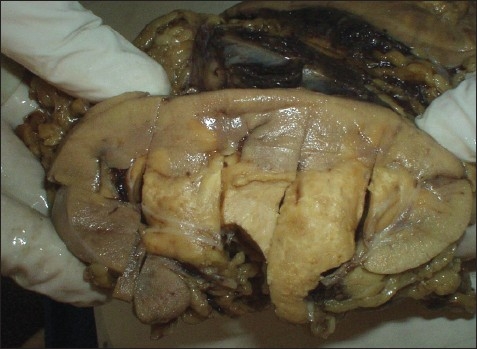
Gross appearance of lesion in renal pelvis. A well-circumscribed mass with a creamy-brownish appearance, hemorrhage foci in cut surface.

**Figure 3:**
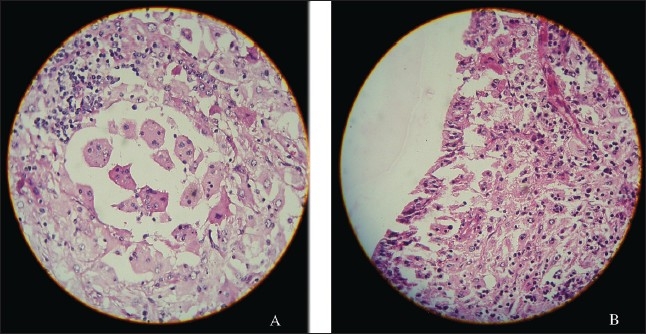
A) Lymphocytes, plasma cells and numerous histiocytes with a large vesicular nuclei and abundant cytoplasm. Many of these histiocytes have within their cytoplasm numerous intact lymphocytes, a feature that has been designated as emperipolesis or lymphocytophagocytosis. (hematoxylin-eosin stain, original magnification ×400). B) Renal pelvis involved by RD, note lymphocytes, plasma cells and numerous histiocytes with emperipolesis. The transitional epithelium is seen on the left of micrograph (hematoxylin-eosin stain, original magnification ×400).

**Figure 4:**
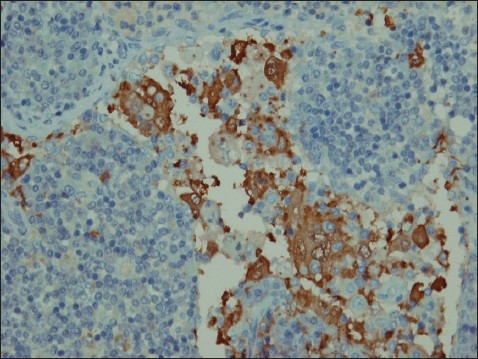
Immunohistochemical staining with S-100 protein revealing strongly stained histiocytes (IHC staining with S-100 protein, original magnification ×800).

A 39-year-old man presented with hematuria. He had no significant previous medical history and a system review was negative. Physical examination revealed a well-nourished individual in apparently good health without peripheral lymphadenopathy, fever or weight loss. Laboratory examination revealed a normal leukocyte count. ESR was significantly increased, up to 115 mm/hour. Hemoglobin was 9.5 g/L, mean corpuscular volume was 78.5 fL, mean corpuscular hemoglobin was 26.2 pg and a peripheral blood smear showed microcytic hypochromic red blood cells. C-reactive protein was 2+ and urine cytology in 3 times showed a mild inflammatory process without malignant cells. All other laboratory studies were normal. An intravenous pyelogram showed a lesion in the pelvis and that the left kidney was larger than right kidney. An abdominal CT scan showed a homogenous low density area involving the left kidney sinus that showed distortion and displacement of the pelvicalyceal system without hydronephrosis (Figure 1). With the presumed diagnosis of renal cell carcinoma, a nephrectomy was done. The macroscopic appearance showed a multinodular tan-yellow hemorrhagic lesion involving the renal sinus and pelvicalyceal system (Figure 2). The lesion merged imperceptibly with the renal parenchyma and abutted the renal capsule. Microscopically, the lesion was composed of sheets of histiocytic cells with abundant granular eosinophilic cytoplasm and ill-defined cytoplasmic borders. The nuclei were variable in size, round, vesicular and with smooth nuclear contours. A background of chronic inflammation was noted, predominantly of a plasmocytic infiltrate. The most prominent feature was the presence of extensive emperipolesis (histiocytes with intracytoplasmic lymphocyte). The infiltrate also involved the renal pelvis and perinephric fat (Figure 3). Immunohistochemical staining with S100 protein revealed strongly stained histiocytes (Figure 4). Both the combined histomorphology and immunohistochemical profile supported the diagnosis of extranodal RDD involving the kidney.

## DISCUSSION

In RDD the key factor that induces both chemotaxis of blood monocytes to the site of the lesion and the differentiation of these monocytes into RDD histiocytes is thought to be M-CSF (macrophage colony-stimulating factor). Monocytes are found in small amounts within the pulp of reactive lymph nodes and the interstitium of other organs that secrete M-CSF, which acts as chemotactic factor to recruit more blood monocytes to the site of the lesion. M-CSF stimulates blood monocytes to differentiate into mature macrophages capable of phagocytosis.^4^ The initial precipitating factor that leads to M-CSF production by resident monocytes, resulting in histiocytosis and potent phagocytosis, is yet to be discovered. This may be part of an immune-mediated disorder or a virus-mediated disorder.

Sinus histiocytosis is a diffuse, lymphoproliferative disorder involving numerous organs that occurs most often in children or young adults, although patients in their 7th decade have also been described. Associated symptoms and signs may be caused by specific organ involvement or may be constitutional, such as fever and weight loss. Laboratory findings include anemia, leukocytosis and serum polyclonal hypergammaglobulinemia. Although early descriptions concluded that nearly every case was marked exclusively by cervical lymph node involvement, other organ systems may be affected including the eye and eyelid, bone, central nervous system, ear, nose, throat, upper respiratory tract, liver, skin, salivary gland and testis. Coexistent lymphadenopathy, especially in retroperitoneal sites such as the para-aorta, iliac and inguinal lymph node chains has been noted in as many as 78% of patients. However, this disorder may also occur primarily, if not exclusively, in an extranodal site such as the facial skin or muscles, orbit, paranasal sinuses^6^ or thyroid.^7^ Kidney involvement is very uncommon, and therefore sinus histiocytosis is not frequently considered in the differential diagnosis of an infiltrative renal mass. Four cases of RDD in kidney cases have been reported with two of them diagnosed with adenocarcinoma of the prostate. None, including the one referred to us, had lymphadenopathy and all were in the same location within the kidney. The patients had been referred for renal masses and urinary conditions. Renal RDD accompanied by prostate adenocarcinoma warrants more study. In a rare case reported by Buchino et al, the kidney was only focally involved with a single small mass in the lower pole that contained an admixture of histiocytes, lymphocyte and plasma cells.^8^ In another case reported by Bechtold et al, a lobular irregularly enlarged kidney with distorted calyces associated with large matted para-aortic lymph nodes was described and the diagnosis was RDD.^9^ Some patients may experience spontaneous regression of the lesion, while others have a chronic course with stable or progressive disease.^10^ Grossly the masses are matted together by prominent perilesional fibrosis. Their cut surface varies from gray to golden yellow, depending on the amount of fat present. Microscopically there is an accumulation of lymphocytes, plasma cells (some containing Russell bodies), and most notably numerous cells of histiocytic appearance with a large vesicular nucleus and abundant clear cytoplasm that may contain large amounts of neutral lipids. Many of these histiocytes have within their cytoplasm numerous intact lymphocytes, a feature that has been designated as emperipolesis or lymphocyte phagocytosis.^11^ Although not specific to RDD, this is a constant feature of RDD and is therefore of great diagnostic significance. Sometimes other cell types are present within the cytoplasm of the histiocytes such as plasma cells and red blood cells. The histiocytes contain cytoplasmic fat and are strongly reactive for S100 protein, but negative for CD1a. The plasma cells show a polyclonal pattern of immunoglobulin expression. The lymphocytes present are an admixture of B and B and T cells.

The differential diagnosis of RDD in kidney includes malignant fibrous histiocytomas and histiocytic proliferations of infectious etiology (the presence of S100 is useful in discriminating these lesions), leukemia or lymphoma, especially when accompanied by lymphadenopathy (absent of emperipolesis and IHC profiles help to correct diagnosis). Other possible differential diagnoses include storage disease, tuberculosis or even renal cell carcinoma, a metastatic tumor such as malignant melanoma. RDD generally has a favorable prognosis, but involvement of a greater number of nodal groups and associated extranodal systems worsens the prognosis.^11^,^12^ A high mortality rate is associated with disseminated nodal disease, the presence of systemic immunological disorders and involvement of unusual sites such as the liver, kidney or lower respiratory tract. No intervention is necessary in most cases, but some patients may undergo surgery. In disseminated aggressive cases, chemotherapy and external may be used.
